# Evaluation of Zimmer® segmental distal femur mega-prostheses: Patient survival, surgical outcomes and functional outcome

**DOI:** 10.1016/j.jbo.2025.100722

**Published:** 2025-11-02

**Authors:** Christina Enciso Holm, Jesper Peter Bömers, Allan Villadsen, Michael Mørk Petersen

**Affiliations:** The Musculoskeletal Tumor Section, The Department of Orthopedic Surgery, Rigshospitalet, University of Copenhagen, Denmark

## Abstract

**Background:**

Modular knee mega-prostheses are considered the method of choice for reconstruction after resection of malignant bone lesions around the knee. The available literature evaluating Zimmer® Segmental mega-prostheses is sparse. The purpose of the present study is to evaluate the use of Zimmer® Segmental mega-prosthesis for reconstruction of the distal femur, following the resection of bone malignancies and aggressive benign bone tumors.

**Material and Methods:**

A retrospective study including 59 consecutive patients (F/M = 35/24), mean age 58 (range 17–86) who underwent reconstructions of the distal femur due to malignant bone lesions (n = 51) or aggressive benign bone tumors (n = 8) from 2017 to 2022. All reconstructions were performed with the most recent Zimmer® Segmental System with the XT Distal Femoral Component. Kaplan-Meier survival analysis was used for evaluation of overall survival. Competing risk analysis was used for assessing cumulative incidence of revision and amputation. Patients were followed until death or end of study (December 31st, 2024). Functional outcome and quality of life was evaluated with Musculoskeletal Tumor Society Score (MSTS), European quality of life − 5 Dimensions score (EQ 5D) and Oxford Knee Score (OKS).

**Results:**

Twenty-eight (n = 28) patients were alive at follow-up. Overall survival after 5-year was 44 % (CI95%: 30–58 %). The risk of implant failure after 1 and 5 years was 8 % (95 %CI 1–16 %) and 12 % (95 %CI 4–20 %) respectively. One patient (2 %) underwent amputation. Mean MSTS score was 17 (57 %) (range 3–30). Mean EQ 5D index score was 0.88 and mean EQ-5D VAS score was 68. OKS demonstrated a mean score of 31.

**Conclusion:**

The Zimmer® Segmental mega-prosthesis in distal femur reconstruction with the improved XT femoral component, demonstrated a relatively low risk of implant failure, and a low risk of amputation. The risk of implant failure was higher among patients who underwent radiotherapy.

## Highlights

• No bumper breakage with second-gen Zimmer® Segmental system.
• 5-year implant failure risk was 12 %.
• 5-year amputation risk was 2 %.
• EQ-5D index score averaged 0.88.
• Mean MSTS score was 17 (57 %).

## 1 Introduction

Lower extremities are the most frequent location for primary and metastatic bone lesions. Most primary bone tumors are located at distal femur and proximal tibia. For decades, resection and reconstruction with mega-prostheses has been the method of choice in the surgical management of primary bone malignancies and aggressive benign bone tumors. This is due to superior function, and similar oncologic outcome compared to amputation [[1], [2], [3], [4], [5], [6], [7], [8]]. Over the last decade, the indications for reconstruction with mega-prostheses has broadened to increasingly include metastatic bone lesions, soft tissue tumors adjacent to bone and also non-malignant bone lesions [3,9,10]. The reasons for the increasing use of mega-prostheses are multiple, including continuous advancements in implant design and a current “off the shelf” availability. It is well described that the risk of complications after insertion of mega-protheses is higher compared to conventional arthroplasties [[11], [12], [13], [14], [15]]. While early reports with mainly fixed-hinge prostheses primarily demonstrated mechanical failures [[16], [17], [18], [19], [20], [21], [22]], later studies evaluating second generation mega-prostheses with rotating-hinge and improved design, describe periprosthetic infection as one of the main causes for revision and, ultimately, secondary amputation, especially around the knee [[2], [3], [4], [5],^23^,^24^]. Additionally, long-term follow up studies have demonstrated that the risk of infection after mega-prostheses remains and increases throughout life [5,18].

Reconstruction of the distal femur, represents significant challenges due to the required restoration of soft tissue, joint stability, and kinetics in often younger patients. Thus, optimizing function and improve long-term implant survival remains a challenge. In a few small reports, implant survival and functional outcome of the Zimmer® Segmental System around the knee has been questioned due to hyperextension, instability and in particular bumper breakage [25,26]. In 2017 a second-generation Zimmer® Segmental system was introduced, enhanced with distal femur XT insert components [27]. The distal femur XT design provides a new designed poly insert and a matching, mating recess on the XT femoral component intentionally to improve hyperextension torque and thus enhance functional outcome and implant survival [27]. Although various reconstruction systems has been evaluated [14,[19], [20], [21],^24^,[28], [29], [30], [31], [32]], the reported literature with the Zimmer® Segmental System, remains sparse [23,25,26]. Our orthopedic oncology Center introduced the Zimmer® Segmental system in 2011 and it gradually became the most frequently used mega-prosthesis until present.

Present study aimed to evaluate 1) implant survival 2) limb survival and 3) functional outcome and quality of life, in patients who underwent resection of the distal femur and reconstruction with the Zimmer® Segmental System.

## 2 Patients and methods

**Fig. 1:**
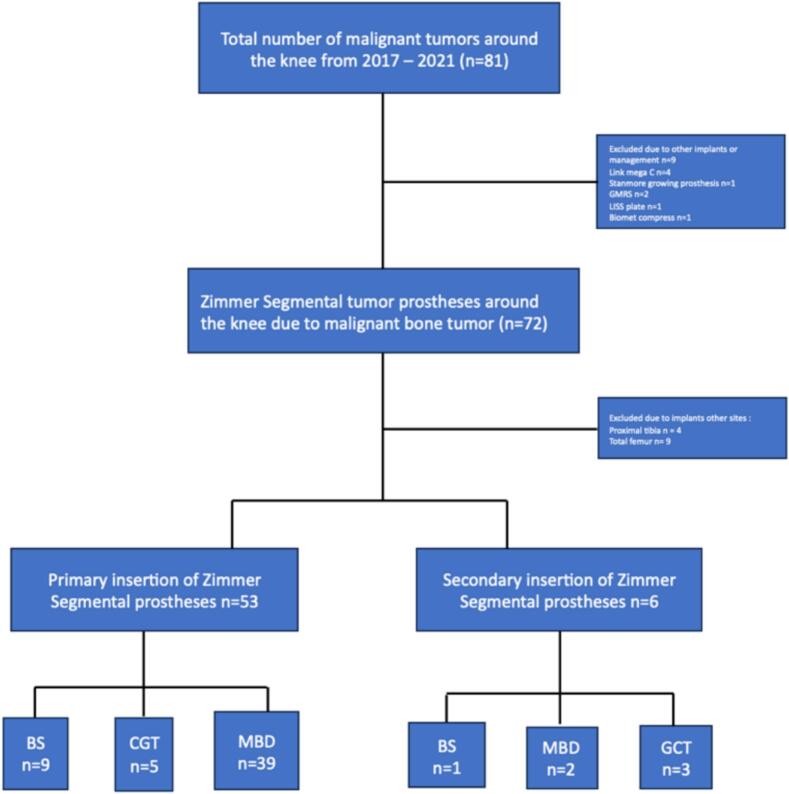
Inclusion of patients *Flowchart illustrating how the cohort was identified and relations between number of implants and patients. A total of n = 59 patients were included. Primary Zimmer Segmental mega-prostheses (n = 53) included: Bone sarcoma (BS), n = 9; Giant cell (GCT) n = 5; metastatic bone disease (MBD) n = 39. Secondary Zimmer segmental prostheses included: Bone sarcoma (BS), n = 1; Giant cell tumor (GCT), n = 3; Metastatic bone disease, n = 2.*

We included a consecutive retrospective cohort of patients who underwent resection of the distal femur due to malignant bone lesions or aggressive benign bone tumors, and reconstruction with The Zimmer® Segmental mega-prostheses system at the Musculoskeletal Tumor section, Department of Orthopedic surgery, Rigshospitalet in Copenhagen between January 1st 2017 and 31st December 2022. At our institution, the indications for resection and reconstruction in patients with MBD are pain relief and regaining function. In GCT the indication is bone lesions, most often recurrences, not amenable to joint-sparing surgery. The indications have remained unchanged during the inclusion period. Due to the social health care system in Denmark, patients with malignant bone lesions and aggressive benign bone tumors, are entitled to free government paid treatment in a public medical care system and will always be referred to one of the two tertiary referral centers covering all highly specialized bone tumor surgeries nationwide. Patients were identified by manually screening our institutional surgical planning system. Inclusion criteria were: patients of all ages who underwent primary or secondary resection and reconstruction with Zimmer® Segmental mega-prostheses due to primary bone sarcoma, aggressive benign bone tumor and metastatic bone disease of the distal femur. Exclusion criteria: patients with bone lesions of the distal femur who underwent other types of surgical treatment or insertion of other types of implants. Thirty patients were excluded due to insertion of other implants or resection at other sites (Fig. 1).

For all analysis, revisions were defined as all unplanned implant-related surgeries. All revisions were recorded from date of surgery until death or end of study. Amputation was defined as amputation of the extremity for all causes, and also analyzed per se. Complications and failures were classified according to the Henderson Failure Mode Classification [33] in five failure types: Type 1 (mechanical failure due to soft tissue problems, such as debridement, peroneal nerve palsy, dislocation of joint (closed reduction), and superficial infections), Type 2 (aseptic loosening), Type 3 (structural failures, such as periprosthetic fractures and hip dislocation requiring surgical treatment), Type 4 (non-mechanical failures, such as deep infection), and Type 5 (tumor progression).

Follow-up included clinical examination, X-ray and evaluation with Musculoskeletal Tumor Society Score (MSTS) [34], Oxford Knee Score (OKS) [35] and European quality of life − 5 Dimensions (EQ-5D) [36]. The MSTS was introduced in 1983 by Enneking et al. and modified in 1993 for evaluation of the functional outcome after treatment of sarcomas. The system estimates from bad to very good with parallel assigned values from 0 to 5 in six categories in the lower extremities: Pain, Function, Emotional acceptance, Supports, Walking, and Gait. Subsequently, the six values are added and divided by the maximum value of 30 and a percent rating is calculated. The OKS is a validated patient-based questionnaire consisting of 12 questions about level of function [37]. Five questions refer to pain (40 %) and seven questions refer to function (60 %) assessing degree of pain and function summed to a total score between 0 and 48 (worst–best). The EQ-5D is commonly used to evaluate patient-reported health-related quality of life by a 5 dimensions questionnaire and is reported with a country specific index score and Visual Analog Scale (VAS) from 0 to 100 (worst – best) [38].

### 2.1 Statistics

Patient demographics were analyzed descriptively and tested for significance using a Chi2-test (categorical variables) and Student *t*-test (continuous variables). The probability of overall survival was estimated using Kaplan–Meier analysis. Log-rank testing was used to compare overall survival between patient sub-groups. A competing risk model (Aalen-Johansson estimate) was used to assess the cumulated incidence of revision and amputation. Death and amputation were defined as competing risks. Gray’s test was used to assess differences between sub-groups. Confidence intervals (CI) are reported as 95 %CI and *p*-values < 0.05 were considered statistically significant. Statistical analysis was performed using software R (R Foundation, Vienna, Austria).

## 3 Results

### 3.1 Patient characteristics

A total of 59 patients (F/M = 35/24) with a mean age of 58 (range 17–86) (Table 1) were included. Primary insertion of Zimmer® Segmental included n = 53 (Fig. 1). Six patients (n = 6) underwent revision with secondary insertion of Zimmer® Segmental including: Mega C (n = 1), Link RHK (n = 1), Link custommade (n = 1), Link (n = 1), GMRS (n = 1), MoM Mutars (n = 1). From patient files we obtained, patient demographics, diagnostics, tumor histology and characteristics, as well as details on surgical procedures, revisions, and death (Table 1). Primary bone sarcoma (BS) included eleven patients (n = 11). Histological diagnosis of primary tumors were mainly osteosarcoma (n = 6) followed by Ewing sarcoma (n = 2), Chondrosarcoma (n = 1) and fibrosarcoma of bone (n = 1), desmoplastic fibroma (n = 1). Metastatic bone disease (MBD) caused the majority of resections and reconstructions (n = 41). Most metastases originated from lung (n = 11) and kidney (n = 7). Non-oncological bone lesions included seven patients (n = 7) with Giant cell tumor (GCT). (Table 2). Patients were followed until death from all causes, or end of study (December 31st, 2024) resulting in a minimum of 3-year follow-up. The Danish Civil Registry [39] ensures no loss of patient survival follow-up.

**Table 1:** Demographic overview of all patients and subgroups consisting of patients suffering from bone sarcomas or giant cell tumor of bone (BS + GCT) and metastatic bone disease (MBD).

	BS + GCT (n = 18)	MBD (n = 41)	*p-value*
Age at surgery (mean (SD))	31.8 (15)	69.5 (9.4)	<0.001
Gender (%)			0.5
Male	9 (15)	15(25)	
Female	9(15)	26 (44)	
Secondary implant (%)	4 (7)	2 (3)	0.06
Surgery time (mean (SD))	221.22 (67.0)	162.20 (67.3)	<0.001
Resection length (mean (SD))	16.2 (4.5)	18.5 (5.9)	0.2
Cemented (%) Unknown	17 (100.0) 1 (2.0)	41(100.0)	NA
Radiotherapy	0	13 (32 %)	NA
Revision (%)	5 (8.0)	8 (14)	0.5
Amputation (%)	1 (2)	0 (0)	0.7
Death (%)	3 (5)	28 (47)	<0.001

**Table 2:** Overview of histological diagnoses of all patients included in the study (n = 59).

Histological diagnoses	Total
*Bone sarcoma*	

Osteosarcoma	6
Ewing sarcoma	2
Chondrosarcoma	1
Desmoplastic fibroma	1
Fibrosarcoma of bone	1

*Benign bone tumor*	

Giant cell tumor	7

*Metastatic bone disease*	

Lung cancer	11
Kidney cancer	7
Breast cancer	4
B cell Lymphoma	4
Myelomatosis	3
Prostata cancer	2
Bladder cancer	2
Lymphoma	2
Cervix cancer	1
Colon cancer	1
Malignant melanoma	1
Neuroendocrine carcinoma	1
planocellulare carcinoma rhinopharynx	1
Unknown	1

### 3.2 Oncological outcome

**Fig. 2:**
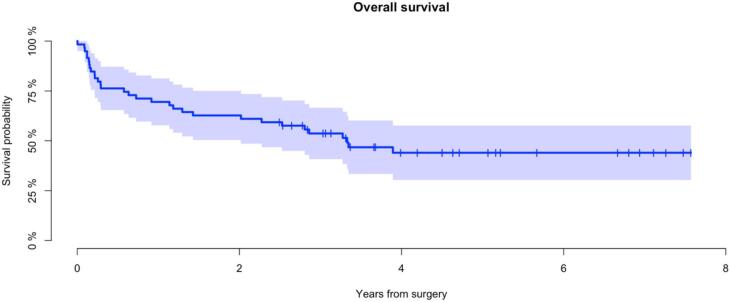
Overall survival all patients (n = 59) *Kaplan-Meier analysis demonstrating the probability of overall survival, all patients (n = 59)*.

**Fig. 3:**
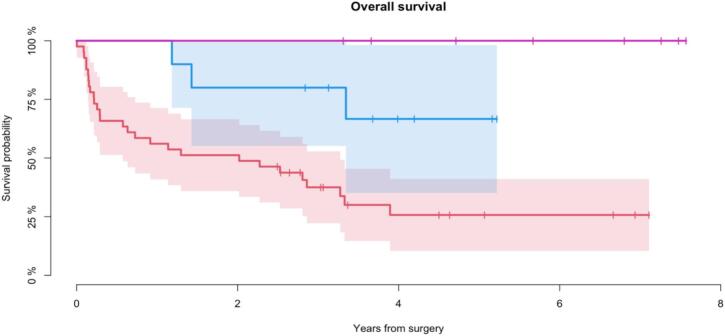
Overall survival within histological subgroups (n = 59) *Kaplan-Meier analysis demonstrating overall survival within subgroups. MBD (n = 41): 26 % (CI95 %: 10*–*41 %). BS (n = 11): 67 % (CI95 %: 35*–*98 %). GCT (n = 7): 100 %. p < 0.001.*

Mean follow-up for all patients was 3 years (range: 1 day–8 years). At latest follow up, 28 patients were alive (47 %). Mean follow-up for patients alive was 5 years (range: 2–8 years). The probability of overall survival after 5 years was 44 % (CI95%: 30–58 %) (Fig. 2). All patients with GCT were alive. The probability of 5-year survival for patients with BS and MBD was 67 % (CI95%: 35–98 %) and 26 % (CI95%: 10–41 %) respectively, demonstrating significantly better survival for patients with BS (p < 0.001) (Fig. 3).

### 3.3 Revisions

**Fig. 4:**
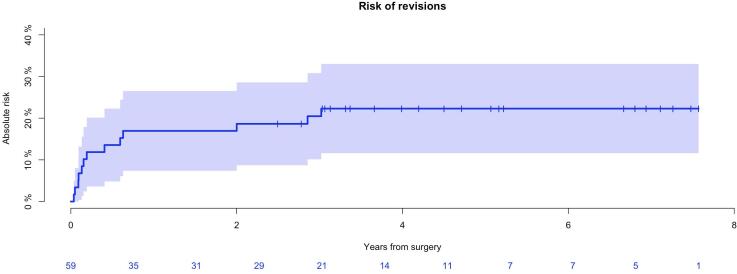
Risk of revisions, all patients (n = 59) *The cumulative incidence of revision after 1 and 5 years demonstrated 17 % (CI95 %: 7*–*27 %) and 22 % (CI95 %: 12*–*33 %).*

Thirteen patients (13/59; 22 %) underwent a total of 21 revisions for all causes (Table 4). Mean time to revision of all causes was 9 months (range 13 days–3 years). The cumulative incidence of revision after 1 and 5 years was 17 % (CI95%: 7–27 %) and 22 % (CI95%: 12–33 %) (Fig. 4) respectively. Primary causes for revision were wound dehiscence (n = 6) followed by structural failures (n = 6) and infection (n = 4) (Table 3 and Table 4). Five patients (5/59; 8 %) had deep infection. The cumulative incidence of deep infection was 9 %(CI95%: 1–16 %) after 1 year and remained unchanged after 5 years. Cumulative incidence of infection in patients with MBD and BS/GCT after 5 year was 7 % (CI95%: 0–15 %) and 11 %(CI95%: 0–26 %) respectively (*p* = 0.6).

**Table 3:** Failure mode classification by anatomical site, according to Henderson et al.

Primary diagnosis (number of revisions)	Type 1 (n = 7)	Type 2 (n = 1)	Type 3 (n = 5)	Type 4 (n = 3)	Type 5 (n = 0)	All types (n = )
	2 months (18 days-5 months)	7.2 months	1.6 year (13 days-3 years)	5.5 month (18 days-11 months)	**−**	
BS (n = 4)	1	1	1	2	−	5
MBD (n = 8)	5		5	2	−	12

**Table 4:** Causes and numbers of all performed implant related revisions.

Revision	Primary implant n = 11	Secondary implant n = 2	Henderson classification
Infection Removal implant Amputation Without removal implant	3 1 1		Type 4 Type 4 −
Aseptic loosening	1		Type 2
Knee joint dislocation	1		Type 3
Periprosthetic fracture	2		Type 3
Aseptic wound dehiscence Septic wound dehiscence	3 2	1	Type 1 −
Loosening bushing	1		Type 3
Joint capsule rupture	1		Type 3
Necrosis patella	1		Type 3
Retropatellar pain, insertion of patella button	1		−
Limb length alignement/extension		1	−

**Fig. 5:**
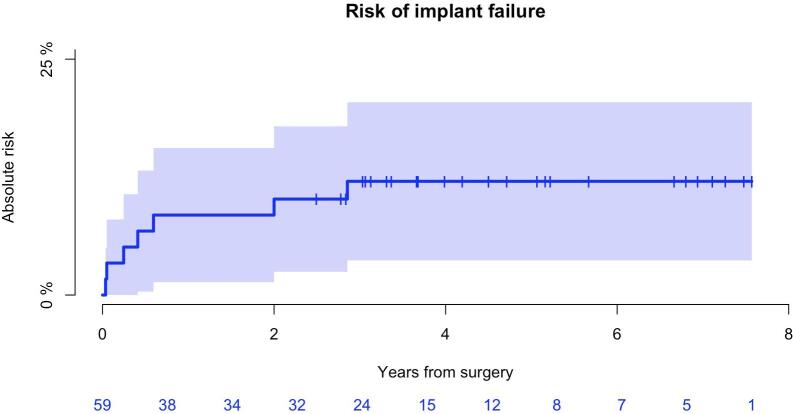
Risk of implant failure all patients (n = 59) *The cumulative incidence of major revisions (implant failure) after 5 years was 12 % (95 %CI: 4*–*20 %)*.

Seven patients (7/59; 12 %) underwent revision with removal of bone anchored implants or amputation, defined as major revisions. Two patients underwent major revision twice. Mean time to major revision was 11 months (range: 13 days – 3 years). Causes were: deep infection (n = 3), necrosis of patella (n = 1), periprosthetic fracture (n = 2), aseptic loosening (n = 1), knee joint dislocation (n = 1), knee joint capsule rupture (n = 1). The cumulative incidence of major revisions (implant failure) after 1 and 5 years was 8 % (95 %CI: 1–16) and 12 % (95 %CI: 4–20 %) respectively (Fig. 5).

**Fig. 6:**
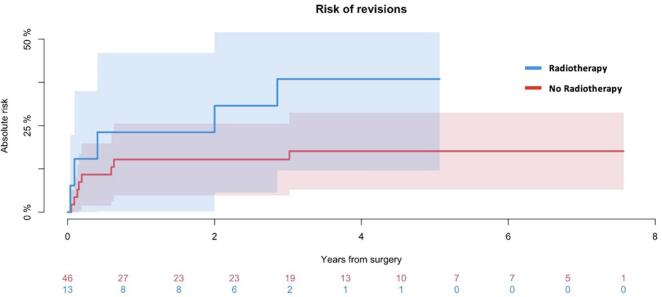
Risk of revision *The cumulative incidence of revisions comparing patients who received RT (n=13) and not (n=46). The risk of revision after 5 years among patients who received RT or not was 39 % (95 %CI: 12*–*65) and 18 % (95 %CI: 7*–*29) respectively (p = 0.1).*

Thirteen patients with MBD (13/59; 22 %) received radiotherapy (RT). Six patients (n = 6) received preoperative RT, six patients (n = 6) received postoperative RT, and one (n = 1) patient received both. Mean time from preoperative RT to surgery was 3 years (range 3 month-10 years). Mean time from surgery to postoperative radiation was 10 month (range 16 days – 3 years). Five patients receiving RT (5/13; 38 %) underwent revision, predominantly major revisions (4/5; 80 %). One patient (n = 1) had aseptic wound dehiscence causing minor revision, one patient (n = 1) had wound dehiscence leading to deep infection and two-stage surgery. Two patients (n = 2) had periprosthetic fractures requiring revision to total femur prostheses, and one (n = 1) patient had wound dehiscence due to necrotic patella leading to removal of implant and consequently arthrodesis. The cumulative incidence of revisions among patients who received RT after 1 and 5 years was 23 % (95 %CI: 0.1–46) and 39 % (95 %CI: 12–65) respectively (*p* = 0.1) (Fig. 6).

### 3.4 Henderson classification

Five patients (5/59; 8 %) (MBD n = 6; BS n = 1) had Type 1 failures (Table 3). Mean time to Type 1 failure was 2 months (range 18 days–5 months). The most common cause for type 1 failure was aseptic wound dehiscence (n = 4), followed by superficial infection (n = 2). One patient (1/59; 2 %) (BS) had type 2 failure due to aseptic loosening after 7.2 months. Five patients (5/59; 8 %) (MBD n = 4; BS = 1) had type 3 failures. Mean time to type 3 failure was 1.6 year (range 13 days–3 year). Two failures were caused by periprosthetic fractures (n = 2) followed by knee joint dislocation (n = 1), necrosis of patella (n = 1), wear (n = 1), and knee joint capsule rupture (n = 1). Three (3/59; 5 %) (MBD n = 2; BS n = 1) had type 4 failure. Mean time to type 4 failure was 5.5 months (range 18 days–11 months). One patient (MBD) had two-stage surgery, one patient had one-stage surgery (MBD), and one patient (BS) with intended two-stage surgery, underwent amputation after stage-one due to lacking effect of treatment. Microbiology is summarized in Table 5.

**Table 5:** Site and causes of deep infection.

Site	No. revisions	Microbiology	Amputation
BS: n = 2	4	*peptoniphilus species* *actinotignum schaaliii,* *anaerobe gram positive stave* *staph.epidermidis*	1
MBD: n = 3	3	*peptoniphilus harei* *staph. Epidermidids* *micorcoccus luteus* *prevotella species* *anaerobe gram positive rods* *e.coli* *pseudomonas aeruginosa* *enterococcus faecium* *corynebacterium tuberculostearicum*	

### 3.5 Limb survival

One patient (1/59; 2 %) underwent amputation. Time to amputation was 1 year. Amputation was caused by deep infection. The cumulative incidence of amputation after 5 year was 2 %(CI95%: 0–5 %).

### 3.6 Functional outcome and quality of life

**Fig. 7:**
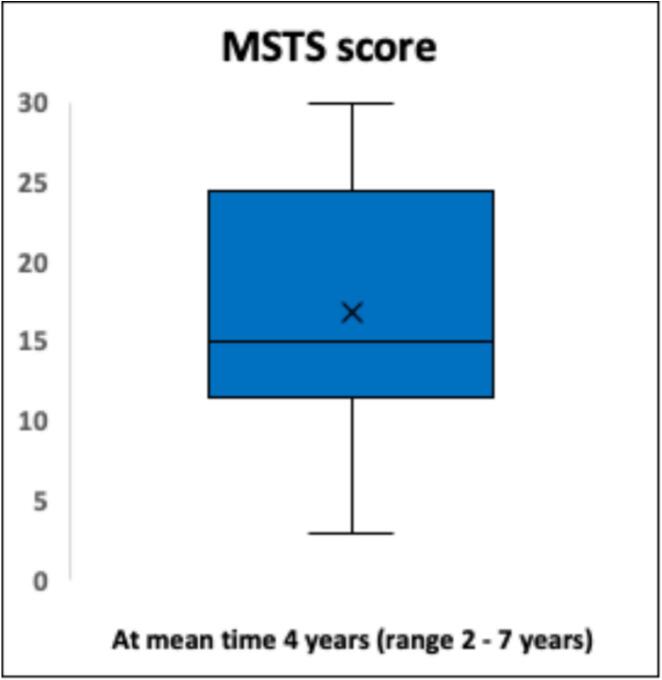
Functional results of 22 patients evaluated with the Musculoskeletal Tumor Society Score (MSTS). *Functional outcome with MSTS score was evaluated in twenty-two patients (22/28; 79 %) after a mean time of 4 years (range 2*–*7 years). Mean MSTS score was 17 (57 %) (range 0*–*100 %).*

**Fig. 8:**
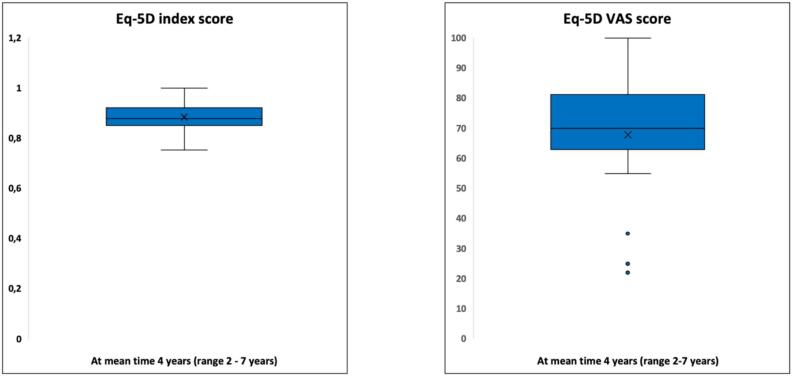
European quality of life − 5 Dimensions (EQ-5D) evaluated in 22 patients *Twenty-two patients (22/28; 79 %) were evaluated with EQ 5D. BS/MBD/GCT = 7/ 9 / 6. Mean EQ 5D index score was 0.88 and mean EQ-5D VAS score was 68.*

**Fig. 9:**
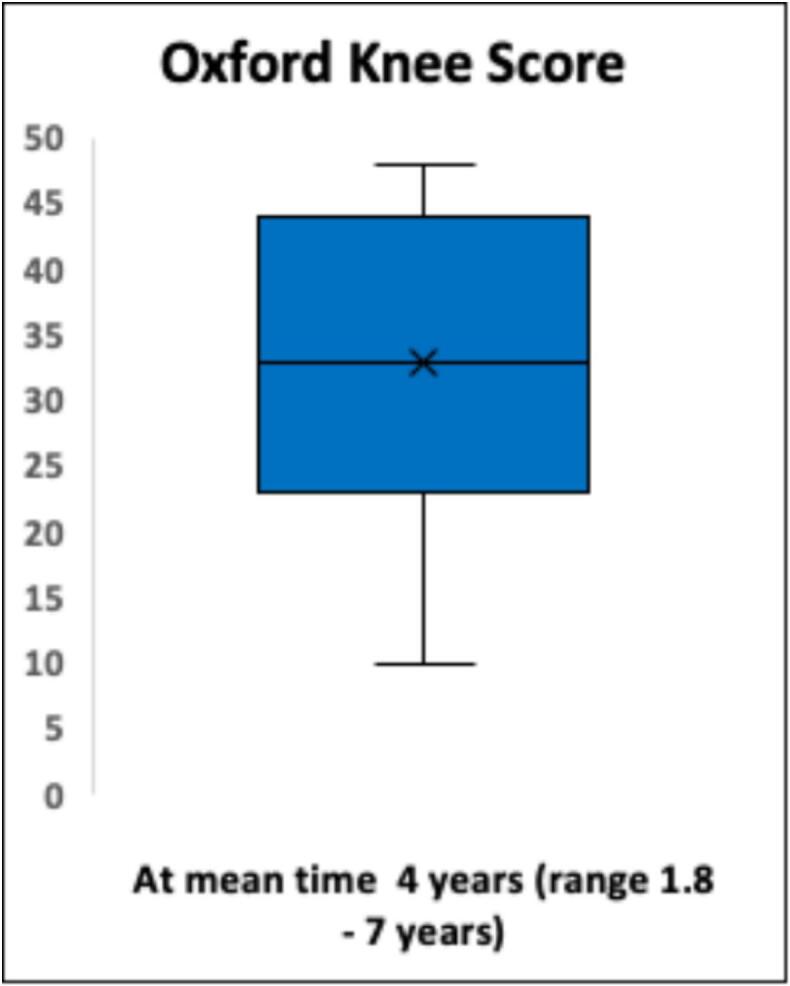
Oxford Knee Score (OKS) evaluated in 18 patients *OKS obtained in 18 patients, demonstrated OKS demonstrated a mean score of 31 [Standard deviation (SD) 11] indicating mild knee problems, minimal pain and limited function.*

Functional outcome and X-ray were evaluated in patients alive and not amputated at the end of study. Twenty-three patients (23/28; 82 %) were evaluated with MSTS score, mean time 4 years (range 2–7 years), BS/MBD/GCT = 7/ 10 / 6. Twenty-two patients (22/28; 79 %) were evaluated with EQ 5D, mean time 4 years (2–7 years), BS/MBD/GCT = 7/ 9 / 6. Eight-teen patients (18/28; 64 %) were evaluated with OKS, mean time 4 years (2 days–7 year), BS/MBD/GCT = 6/ 8 /4. Mean MSTS score was 17 (57 %) (range 0–100 %) (Fig. 7). Mean EQ 5D index score was 0.88, mean EQ-5D VAS score was 68 (Fig. 8). OKS demonstrated a mean score of 31 [Standard deviation (SD) 11] (Fig. 9).

## 4 Discussion

The probability of overall patient survival after 5 years was 44 % (95 % CI: 30–58 %). In total, thirteen patients (22 %) underwent revision, with a mean time of 9 months (range 13 days–3 years). The 5-year incidence of revisions for all causes was 22 % with wound dehiscence and structural failure causing most complications. The 5-year incidence of implant failure was 12 %. The incidence of implant failure after 5 year among patients who underwent RT was 39 %. The 5-year incidence of amputation was 2 %. Deep infection occurred in 8 % of patients. The 5-year incidence of deep infection was 8 %.

Review of recent literature evaluating implant survival of mega-prostheses around the knee due to malignancies, suggests that our results are comparable [28,[40], [41], [42]]. We have previously reported implant survival of our first and second generation mega-prostheses in the lower extremities [23]. The risk of implant failure is slightly favorably in the present cohort; we consider this to reflect the exclusion of proximal tibia and total femur reconstructions in the present cohort, although it could result from the enhanced design of the prostheses. Zhang et al. [42] reported lower implant survival after 5 years (77 %) primarily due to aseptic loosening of the Cemented LINK ENDO-MODEL rotating hinge knee prostheses (Waldemar Link GmbH & Co.KG, Hamburg, Germany). With similar follow up time, only one patient in the present cohort presented with aseptic loosening despite a mean resection length of 18 cm and cementation of all stems. Distal femur reconstructions are well-known to be at risk for aseptic loosening due to the torsional forces in the bone-implant interface [20,43,44]. We suggest that the substantial rotation possibilities with greater range of motion in the Zimmer® Segmental prostheses, reduces torsional forces and subsequently aseptic loosening although our result also could reflect mid-term follow-up. To avoid aseptic loosening and improve osteointegration at the bone-prosthetic interface, the Compress® Compliant Pre‐Stress (CPS) for reconstruction after distal femur resection has been suggested, particularly in pediatric patients due to its expandable potential. Despite proven osseointegration [45,46], Tanaka et al. [46] recently reported a relatively high revision rate. At our institution, the Compress®/OSS is not offered by standard and none of the patients in present study received Compress®. The indications at our center would primarily include very high resections in pediatric patients with BS. However, since the implant is uncemented, our consern is the risk of greater bone loss in case of revision, compared with a cemented implant. Furthermore, the adapter required to pair the Compress® with the Zimmer® Segmental system is custom-made, hence time-consuming given the often limited time available.

Pala et al. [28] reported 5-year implant survival of 70 % of the Global Modular Replacement System (GMRS®) around the knee. However, their cohort comprised 60 proximal tibia resections who suffered the highest proportion of complications. Geiger et al. [41] compared primary and secondary reconstructions of distal femur malignant bone lesions from 1980 to 2018. Despite comparable cohorts, the low proportion of secondary implants in the present cohort did not justify any sufficient sub-group comparison. The cumulative survival in primary implants after 5 years reported by Geiger et al. was 78 %. Although the reporting of results is not directly comparable, present incidence of 12 % after 5 years, indicate a lower risk of implant failure. The difference likely reflects the use of first-generation fixed-hinge mega-prostheses in Geiger et al. [41], as opposed to the modern design in present cohort. Bus et al. [40] evaluated the Modular Universal Tumor and Revision System (MUTARS®) between 1995 and 2010 and reported 5-year incidences of implant failure of 17 % due to mechanical failure and 8 % due to deep infection. In present study, the majority of implant failure were likewise mechanical (types 1–3), followed by deep infection (type 4). Given that most patients with implant failure in present cohort received RT, it is plausible that RT contributed to these outcomes. This is supported by the present demonstrated tendency towards a higher risk of revision among irradiated patients. The association between RT and a higher risk of implant failure has previously been reported by Theil et al. [47], who found a higher rate of complications among patients receiving postoperative rather than preoperative RT. In present cohort, most patients with implant failure received preoperative RT; however, the limited number of irradiated patients precludes causality.

While deep infection is considered to cause most revisions after insertion of modern mega-prostheses in general [2,5,33], present findings demonstrated higher incidence of soft-tissue failure (type 1) and structural failures (type 3) compared to deep infection (type 4). Viewing the literature it remains inconclusive if mechanical complications, in particular structural failures, exceeds the risk of infections in mega-prostheses around the knee [24,31,[40], [41], [42],^44^,^48^]. However, the different outcomes seems partly due to inclusion of various generations of mega-prostheses combined with heterogeneous reporting: Pala et al. [28] did not report any structural failures and other authors do not define wear of polyethylene parts as a complication, in contrast to the present study [41,49]. We believe that wear of polyethylene bushings should be considered a complication since replacement of bushings and other polyethylene parts has been demonstrated to pose a risk for secondary infection [50]. In current study only one patient underwent revision do to wear of bushing, most likely due to mid-term follow-up, although we note that no patients had early bumper breakage as demonstrated in previous reports [25,26]. Furthermore, despite the suggested classification by Henderson et al. [33], there is no consensus on the extent of complications classified as structural failure modes, which therefor remains diverse and limiting adequately interstudy comparison and interpretation.

In present study, deep infection presented in five implants (8 %) and the 5-year risk of infection was 9 %. These findings compare favorably to previous reports of mega-prostheses around the knee, ranging from 11 % to 15 % [28,40,42,50,51]. Geiger et al. [41] also reported a relatively low rate of infections (6 %), indicating that distal femur reconstructions poses a lower risk of infection compared to proximal tibia as also suggested in current study. Nevertheless, since deep infection is one of the most frequently reported complications [5,33,43,52] and most often leads to reoperation and ultimately amputation as demonstrated in present cohort, it is considered to be the most devastating complication with greatest impact for the patients. We did not detect any local recurrences (type 5), most likely due to mid-term follow-up time and by the inclusion of mainly MBD.

The average MSTS score was 57 %. Despite various results in comparable reports ranging from 70 % to 83 % [24,28,31,32,42,53], our results compares fairly poor. Smolle et al. [2] found a tendency towards poorer functional outcomes with larger resections and also a correlation between larger implants and complications which further can implicate functional outcome. This is supported by Innocenti et al. [31], who found that MSTS decreased progressively with multiple revisions [31]. We suggest that our results partly reflect large resections (mean 18 cm.). Furthermore, the inclusion of patients in general poor health condition with MBD yields a higher mean age compared to other studies [24,28,32] which also has been demonstrated to affect functional outcome [48]. Also, our MSTS is undoubtedly also a reflection of medium follow-up time. OKS has been suggested as one of the best-performing PROMS in patients undergoing knee replacement [37], although extremely rarely used in the evaluation of mega-prostheses due to malignant bone lesions [48]. Our mean score indicated mild knee symptoms with some knee discomfort. Toepfer et al. [48] evaluated patients with distal femur and reported a mean OKS of 31 ± 11 hence very aligned with our results. Lastly, evaluation of quality of life by EQ-5D, yielded a mean index score of 0.88 and VAS score 68, indicating a relatively high quality of life. However, quality of life assessment in patients with MBD is often confounded by adverse effects to parallel oncological treatment, and general poor health condition which should be taken into consideration when interpretating results.

## 5 Limitations

The study is a retrospective non-randomized study with the inherent limitations. Furthermore, due to the inclusion of BS, GCT and MBD, the cohort is very heterogeneous with regards to diagnosis, staging, oncologic treatment and general health condition which unavoidably will affect outcome. However, we included all patients available with none lost to follow-up and limited missing data. Our cohort should therefore in principle be representative to the population of interest and aim of the study. Despite the proposed classification system from Henderson et al. [15], inter-study comparisons of studies evaluating mega-prostheses remains a challenge due to heterogeneous and inconsistent reporting, as a result of varying methodologies [50,54]. Nevertheless, by having a consecutive cohort with no loss to follow up and using the methodology of the present study, including the use of competing risk analysis, we believe that our results provide a true reflection of the population of interest.

## 6 Conclusion

At a minimum of 3 years, the Zimmer® Segmental mega-prosthesis in distal femur reconstruction with the improved XT femoral component, demonstrated a relatively low risk of implant failure, and a low risk of amputation. Wound dehiscence was the most frequent complication. The risk of implant failure was higher among patients who underwent radiotherapy. Quality of life and functional outcomes were acceptable.

## CRediT authorship contribution statement

**Christina Enciso Holm:** Conceptualization, Data curation, Formal analysis, Investigation, Methodology, Project administration, Writing – original draft, Writing – review & editing. **Jesper Peter Bömers:** Data curation, Investigation, Review & editing. **Allan Villadsen:** Data curation. **Michael Mørk Petersen:** Conceptualization, Methodology, Project administration, Supervision, Writing – review & editing.

## Declaration of competing interest

The authors declare that they have no known competing financial interests or personal relationships that could have appeared to influence the work reported in this paper.
